# Correcting for 16S rRNA gene copy numbers in microbiome surveys remains an unsolved problem

**DOI:** 10.1186/s40168-018-0420-9

**Published:** 2018-02-26

**Authors:** Stilianos Louca, Michael Doebeli, Laura Wegener Parfrey

**Affiliations:** 1 Biodiversity Research Centre, University of British Columbia, Vancouver, Canada; 2 Department of Zoology, University of British Columbia, Vancouver, Canada; 3 Department of Mathematics, University of British Columbia, Vancouver, Canada; 4 Department of Botany, University of British Columbia, Vancouver, Canada

**Keywords:** 16S rRNA, Gene copy number, Microbiome surveys, Phylogenetic reconstruction

## Abstract

The 16S ribosomal RNA gene is the most widely used marker gene in microbial ecology. Counts of 16S sequence variants, often in PCR amplicons, are used to estimate proportions of bacterial and archaeal taxa in microbial communities. Because different organisms contain different 16S gene copy numbers (GCNs), sequence variant counts are biased towards clades with greater GCNs. Several tools have recently been developed for predicting GCNs using phylogenetic methods and based on sequenced genomes, in order to correct for these biases. However, the accuracy of those predictions has not been independently assessed. Here, we systematically evaluate the predictability of 16S GCNs across bacterial and archaeal clades, based on ∼ 6,800 public sequenced genomes and using several phylogenetic methods. Further, we assess the accuracy of GCNs predicted by three recently published tools (PICRUSt, CopyRighter, and PAPRICA) over a wide range of taxa and for 635 microbial communities from varied environments. We find that regardless of the phylogenetic method tested, 16S GCNs could only be accurately predicted for a limited fraction of taxa, namely taxa with closely to moderately related representatives (≲15% divergence in the 16S rRNA gene). Consistent with this observation, we find that all considered tools exhibit low predictive accuracy when evaluated against completely sequenced genomes, in some cases explaining less than 10% of the variance. Substantial disagreement was also observed between tools (*R*^2^<0.5) for the majority of tested microbial communities. The nearest sequenced taxon index (NSTI) of microbial communities, i.e., the average distance to a sequenced genome, was a strong predictor for the agreement between GCN prediction tools on non-animal-associated samples, but only a moderate predictor for animal-associated samples. We recommend against correcting for 16S GCNs in microbiome surveys by default, unless OTUs are sufficiently closely related to sequenced genomes or unless a need for true OTU proportions warrants the additional noise introduced, so that community profiles remain interpretable and comparable between studies.

**Electronic supplementary material:**

The online version of this article (10.1186/s40168-018-0420-9) contains supplementary material, which is available to authorized users.

## Introduction

Amplicon sequencing of the 16S ribosomal RNA (rRNA) gene is widely used for estimating the composition of bacterial and archaeal communities. Global microbial diversity initiatives, including the Human Microbiome Project [1], the Earth Microbiome Project [2], and the Tara Oceans global ocean survey [3], use the 16S rRNA gene to determine which microbes are present by matching 16S rRNA sequence variants to reference databases like SILVA [4] and estimate the proportions of taxa based on relative read counts. Many bacteria and archaea, however, have more than one copy of the 16S gene, which leads to biased cell count estimates when the latter are estimated solely based on 16S rRNA read counts [5]. This has led to efforts to predict the distribution of 16S gene copy numbers (GCNs) across clades based on available sequenced genomes, in order to then correct 16S rRNA read counts to account for variable 16S GCNs in cells [5–8]. These corrections can substantially affect community profiles and diversity patterns, since some clades have over 10 copies of the 16S rRNA gene [5, 7]. It is thus important to carefully evaluate the accuracy [9] of predicted 16S GCNs across the wide range of microbial clades encountered in microbiome surveys. Inaccurate prediction of 16S GCNs can introduce substantial noise to community profiles, which can be worse than the original GCN-related biases, particularly when prediction methods differ between studies.

An accurate prediction of 16S GCNs relies heavily on the assumption that 16S GCNs are sufficiently phylogenetically conserved. That is, 16S GCNs must be autocorrelated among related taxa at least across phylogenetic distances typically covered by available sequenced genomes [10]. Kembel et al. [5] found that 16S GCN exhibits a strong phylogenetic signal, as measured by Blomberg’s *K* statistic [11], and concluded that 16S GCNs may be predictable based on phylogenetic placement with respect to genomes with known 16S GCN. A similar conclusion was reached independently by Angly et al. [7], based on a strong phylogenetic signal as measured by Pagel’s *λ* [12]. However, neither Blomberg’s *K* nor Pagel’s *λ* make any statement about time scales (nor phylogenetic scales) over which traits vary. While 16S GCN variation is relatively rare within species, variation increases with taxonomic distance [13] and this may lead to inaccurate predictions for the many clades which are distant from sequenced genomes. To date, no independent evaluation of existing 16S GCN prediction tools has been published.

To resolve these uncertainties, we assessed the phylogenetic autocorrelation of 16S GCNs across bacteria and archaea (prokaryota) in a phylogenetic tree comprising ∼ 570,000 OTUs (99% similarity in 16S rRNA), based on ∼ 6800 quality-checked complete sequenced genomes. The tree was constructed from sequences in SILVA and partly constrained using SILVA’s taxonomic annotations. We predicted 16S GCNs using several common phylogenetic reconstruction methods and examined the accuracy achieved by each method for OTUs in the SILVA-derived tree. We assessed the predictive accuracy as a function of an OTU’s nearest-sequenced-taxon-distance (NSTD), that is, the minimum phylogenetic distance (mean nucleotide substitutions per site) of the OTU to the nearest sequenced genome. We note that the average NSTD for a particular microbial community, weighted by OTU frequencies, is known as its nearest sequenced taxon index (NSTI; [6]). Further, we systematically assessed the predictive accuracy of three recent tools for correcting 16S GCNs in microbiome surveys, PICRUSt [6], CopyRighter [7], and PAPRICA [8], which together have been cited over 1000 times. While PICRUSt and PAPRICA were mainly designed to predict community gene content based on 16S amplicon sequences, they automatically include an intermediate step for predicting and correcting for 16S GCNs. We evaluate the accuracy of these tools using the known GCNs of the aforementioned sequenced genomes, as a function of a genome’s NSTD. To further evaluate these tools under more realistic scenarios, we also compare all tools to each other for OTUs in 635 prokaryotic communities sampled from diverse natural environments, including the ocean, lakes, hot springs, soil, bioreactors, and animal guts. We find that 16S GCNs are moderately phylogenetically conserved and that prediction of 16S GCNs for the large number of clades without sequenced genomes from close relatives will generally be inaccurate. This conclusion is verified by our finding of low predictive accuracies by CopyRighter, PICRUSt, and PAPRICA, both for the sequenced genomes as well as when compared to each other on microbiomes. Using phylogenetically predicted 16S GCNs to correct 16S read counts in microbiome surveys, as previously suggested [5–7], worked well only for a small number of microbiomes.

## Results and discussion

### How predictable are 16S GCNs from phylogeny?

**Fig. 1:**
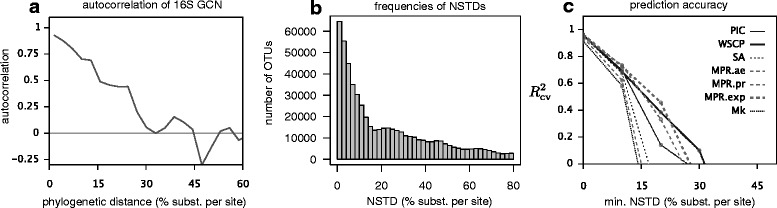
Phylogenetic signal of 16S gene copy numbers (SILVA-derived tree). **a** Pearson autocorrelation function of 16S GCNs depending on phylogenetic distance between tip pairs, estimated based on ∼ 6,800 sequenced genomes. **b** Distances of tips in the SILVA-derived tree to the nearest sequenced genome. Each bar spans an NSTD interval of 2%. **c** Cross-validated coefficients of determination ($R^{2}_{\text {cv}}$) for 16S GCNs predicted on the SILVA-derived tree and depending on the minimum NSTD of the tips tested, for various ancestral state reconstruction algorithms (PIC: phylogenetic independent contrasts, WSCP: weighted squared-change parsimony, SA: subtree averaging, MPR: maximum parsimony reconstruction, Mk: continuous-time Markov chain model with equal-rates transition matrix). MPR transition costs either increased exponentially with transition size (“exp”), proportionally to transition size (“pr”), or were equal for all transitions (“ae”). For analogous results using the original SILVA tree, see Additional file 1: Figure S1

We found that the autocorrelation function of 16S GCNs, that is the correlation between the GCNs of two randomly picked OTUs at a certain phylogenetic distance, decays moderately with increasing phylogenetic distance (Fig. 1a), dropping below 0.5 at a phylogenetic distance of ∼ 15% and to zero at a phylogenetic distance of ∼ 30% (nucleotide substitutions per site in the 16S gene). Hence, predictions of 16S GCNs are expected to be inaccurate for clades with an NSTD greater than about 15% and close to random for clades with an NSTD greater than about 30%. To explicitly test this conclusion, we predicted 16S GCNs for randomly chosen tips of our SILVA-derived tree and compared these predictions to the GCNs known from complete sequenced genomes, where possible. We considered the following common ancestral state reconstruction algorithms for predicting GCNs: Sankoff’s maximum-parsimony with various transition costs [14], maximum-likelihood of Mk models with rerooting (equal rates model), weighted-squared-change parsimony [15], phylogenetic independent contrasts (PIC) [16], and subtree averaging (arithmetic average of GCNs across descending tips). CopyRighter and PICRUSt use PIC, while PAPRICA uses subtree averaging. We measured the accuracy of each method using the cross-validated coefficient of determination $\left (R^{2}_{\text {cv}}\right)$ [17]. The $R^{2}_{\text {cv}}$ corresponds to the fraction of variance explained by a reconstruction algorithm, when tested against a separate set of randomly chosen sequenced genomes (“test set”) than those used for state reconstruction (“training set”). We assessed the $R^{2}_{\text {cv}}$ depending on the NSTD of the test set, that is, the phylogenetic distance between the test set and the training set. We observed that all prediction methods only achieved high accuracies ($R^{2}\gtrsim 0.6$) for NSTDs below about 15–30% depending on the method (Fig. 1c), consistent with our expectations based on the autocorrelation function. At NSTDs greater than ∼ 40%, the $R^{2}_{\text {cv}}$ drops below zero for all methods. Maximum parsimony with exponentially weighted transition costs (“MPR.exp”) was generally the best performing method, while Mk model maximum-likelihood was the worst method (Fig. 1c).

Within the SILVA-derived tree, about 49% of OTUs have an NSTD greater than 15% and about 30% of OTUs have an NSTD greater than 30% (Fig. 1b). We note that natural microbial communities are generally not a purely random subsample of SILVA, since SILVA overrepresents organisms of clinical or industrial interest, and these organisms are generally expected to have low NSTDs. Further, it is likely that a much larger number of prokaryotes not yet included in SILVA, such as from recently discovered or yet undiscovered phyla [18, 19], has NSTDs greater than 30%. Consequently, predictions of 16S GCNs based on sequenced genomes alone are expected to be inaccurate for the majority of extant prokaryotic clades in natural environments. We note that, in principle, errors in tree topology and branch lengths could contribute to a reduced predictive accuracy of phylogenetic reconstruction tools (Fig. 1c). As we show below, however, our expectations on the limited predictability of GCNs are verified in several additional and independent analyses, as well as using the original SILVA tree (Additional file 1: Figure S1).

### Assessment of 3rd party prediction tools

The preceding analysis suggests that phylogenetic prediction of 16S GCNs based on available sequenced genomes is bound to be inaccurate for a substantial number of prokaryotic clades, especially those with only a few sequenced representatives. This finding casts doubts over claims that 16S GCNs can be accurately predicted for typical environmental 16S sequences [5] and that 16S GCN corrections should be applied systematically to every microbiome survey [7]. We thus tested the predictive accuracy of three recently published tools, PICRUSt v1.1.1 [6], CopyRighter v0.46 [7], and PAPRICA v0.4.0b [8]. We performed two types of tests. In the first test, we compared the GCNs of the aforementioned sequenced genomes to the GCNs predicted by each tool based on a genome’s 16S sequence. Because many of these genomes were also used as input to CopyRighter, PICRUSt, and PAPRICA for model calibration in the original publications (“calibration genomes”), or are closely related to those calibration genomes, we evaluate the predictive accuracy of each tool depending on a genome’s distance (NSTD) from the tool’s calibration genomes. In the second test, we compared the predictions of each tool to those of the other two tools, for all OTUs in the Greengenes 16S rRNA database [20] as well as for prokaryotic OTUs found in 635 microbiomes from a diverse range of environments. For each tool, a slightly different approach was taken depending on the tool’s particular design. For PICRUSt and CopyRighter, we used their precomputed lookup tables listing predicted 16S GCNs for entries in Greengenes and mapped genomes (first test) as well as OTUs (second test) to Greengenes entries (at ≥ 99% similarity) to obtain the corresponding GCN predictions. For PAPRICA, we used the 16S rRNA sequences of the genomes or OTUs as input to predict their GCNs through the PAPRICA pipeline.

**Fig. 2:**
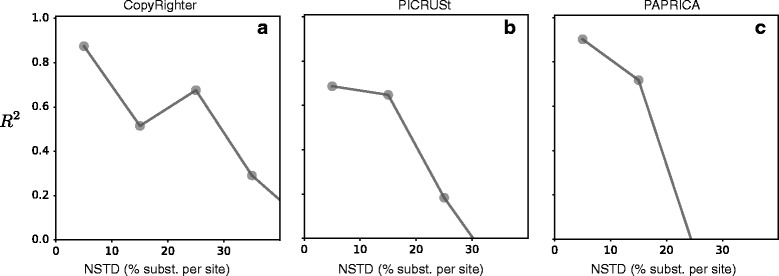
Evaluation of GCN prediction tools on genomes with known GCNs. Accuracy of GCN predictions by CopyRighter (**a**; [7]), PICRUSt (**b**; [6]), and PAPRICA (**c**; [8]) for sequenced genomes, as a function of the genome’s NSTD. NSTDs were calculated separately for each tool, based on the set of genomes used to calibrate the tool by its authors. Accuracy was measured in terms of the coefficient of determination, i.e. the fraction of variance in true GCNs explained by each tool (*R*^2^). Genomes were binned into equally sized NSTD intervals (i.e., 0–10%, 10–20% etc.), and the *R*^2^ was calculated separately for genomes in each bin (one plotted point per bin). Only bins with at least 10 genomes are shown

**Fig. 3:**
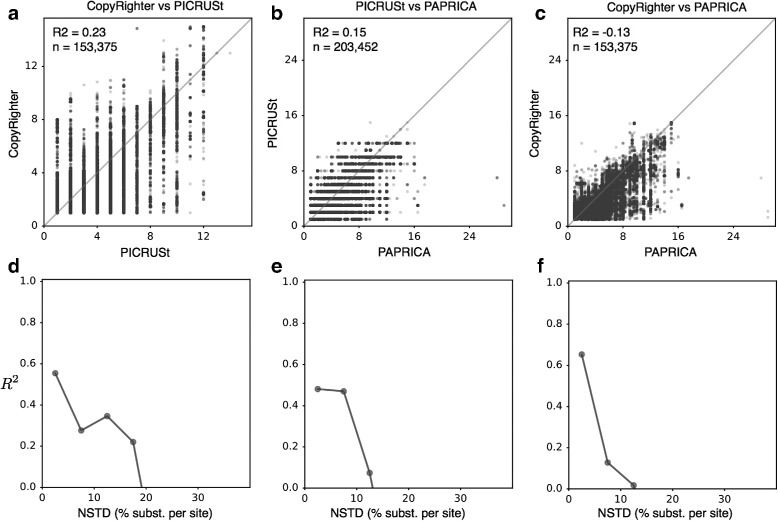
Comparisons of 16S GCN predictions between tools across Greengenes. **a** 16S GCNs predicted by CopyRighter (vertical axis; [7]) and PICRUSt (horizontal axis; [6]) across OTUs (99% similarity) in the Greengenes 16S rRNA reference database (release May 2013; [20]). One point per OTU. **b** Comparison of predicted 16S GCNs by PICRUSt and PAPRICA, similarly to (**a**). **c** Comparison of predicted 16S GCNs by CopyRighter and PAPRICA, similarly to (**a**). Diagonal lines are shown for reference. Fractions of explained variance (*R*^2^, *X*-axis explaining *Y*-axis) and the number of considered OTUs (*n*) are written in each figure. **d–f** Fractions of explained variance (*R*^2^) as a function of an OTU’s NSTD, for each compared pair of tools in **a–c**. OTUs were binned into equally sized NSTD intervals (i.e., 0–5%, 5–10% etc.), and the *R*^2^ was calculated separately for OTUs in each bin (one plotted point per bin). Only bins with at least 10 OTUs are shown

We find that the predictive accuracy of all three tools, evaluated on the sequenced genomes and measured in terms of the fraction of explained variance of true GCNs (*R*^2^), generally decreases with a genome’s NSTD (Fig. 2). Specifically, while accuracy is moderate to high at low NSTDs (*R*^2^ > 0.6 for NSTDs < 10%), the *R*^2^ drops below 0.5 for genomes with NSTDs above 30%. In fact, for PICRUSt and PAPRICA, the *R*^2^ even becomes negative for NSTDs above 30%, which is worse than if the average GCN over all genomes had been used as prediction. We also find poor agreement between the predictions of different tools, when compared to each other across entries in the Greengenes database. When evaluated over the entire Greengenes database, GCNs predicted by any tool explained at most 25% of the variance in the predictions of other tools (*R*^2^<0.25; Fig. 3a–c). It is noteworthy that CopyRighter and PICRUSt use the same set of input genomes (∼ 3000 genomes from the Integrated Microbial Genomes database; [21]) and similar reference trees (Greengenes releases October 2012 and May 2013, respectively), and yet, GCN predictions differ substantially between CopyRighter and PICRUSt (*R*^2^=0.23; Fig. 3a). When we considered the agreement between tools depending on an OTU’s NSTD (Fig. 3d–f), we found that the *R*^2^ decreases rapidly with increasing NSTD and becomes negative at NSTDs below 20%. We also found that the frequency distributions of 16S GCNs predicted across Greengenes (Additional file 1: Figure S1) as well as across the genomes (Additional file 1: Figure S4) differ substantially between tools. For example, CopyRighter, PICRUSt, and PAPRICA predict that the most common GCN across genomes is 1, 3, and 2, respectively. As seen in Fig. 3a, c, CopyRighter indeed appears to underestimate GCNs when compared to PICRUSt and PAPRICA.

**Fig. 4:**
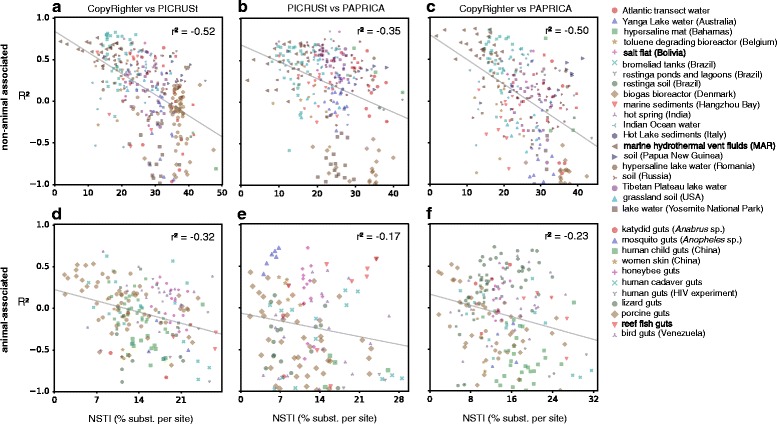
Agreement of GCN prediction tools in microbial communities, depending on the NSTI. **a** Agreement between 16S GCNs predicted by CopyRighter and PICRUSt (in terms of the fraction of variance in the former explained by the latter, *R*^2^) for non-animal-associated microbial communities, compared to the nearest sequenced taxon index (NSTI) of each community. Each point represents the *R*^2^ and the NSTI of one microbial community sample. **b, c** Similar to **a**, but comparing PICRUSt to PAPRICA (**b**) and CopyRighter to PAPRICA (**c**). **d–f** Similar to **a–c**, but showing animal-associated samples. In all figures, linear regression lines are shown for reference. Pearson correlations between *R*^2^ and NSTI (*r*^2^, written in each figure) were statistically significant (*P*<0.05) in all cases. Points are shaped and colored according to the original study, as listed in the legend. Note the negative relationship between a community’s NSTI and the pairwise agreement of GCN prediction tools for that community. For a similar figure showing the spread of NSTDs in each sample, see Additional file 1: Figures S5. For detailed comparisons between tools on individual samples see Additional file 1: Figures S6 and S7

When we compared CopyRighter, PICRUSt, and PAPRICA for OTUs detected in any of the 635 samples, we found that the tools only agreed moderately to poorly with each other for the majority of the samples. Specifically, for any given pair of tools (CopyRighter vs. PICRUSt, PICRUSt vs. PAPRICA, or CopyRighter vs. PAPRICA), the fraction of variance in predictions of the 1st tool that was explained by predictions of the 2nd tool (*R*^2^) was below 0.5 for over 84% of the samples and below 0.1 for over 55% of the samples (Fig. 4). In many cases, the agreement between tools was even worse than if predictions were uncorrelated between tools (*R*^2^<0). A negative *R*^2^ may be indicative of “overfitting” during extrapolation of GCNs to OTUs with large NSTDs. The worst agreement was found between PICRUSt and PAPRICA (mean *R*^2^=− 0.70), while the best (but still bad) agreement was found between CopyRighter and PICRUSt (mean *R*^2^=− 0.41). Even when we only considered animal-associated samples (e.g., from human guts or skin), which are considered better studied than other environments and generally have lower NSTIs (weighted mean NSTD of considered OTUs), we found frequent bad agreements between tools. One explanation is that even in human-associated microbiomes, many OTUs had large NSTDs and were driving overall predictive accuracy down. In fact, we find that the poor agreement between tools in most samples is not driven solely by a few outlier OTUs but is a reflection of moderate to poor agreement for a large number of OTUs in each sample (Additional file 1: Figures S5, S8, and S9). The agreement between tools generally decreased for larger NSTIs, although this trend became much more pronounced when we considered animal-associated samples separately from non-animal-associated samples. The strongest trend was observed in non-animal-associated samples, where the *R*^2^ and NSTI exhibited significant (*P*<0.05) Pearson correlations between − 0.35 and − 0.52, depending on the tools compared (Fig. 4a–c). In animal-associated samples, the *R*^2^ and NSTI exhibited significant Pearson correlations between − 0.17 and − 0.32 (Fig. 4d–f). The above observations are consistent with our expectation that GCN predictions will only be accurate for a small fraction of naturally occurring microbiomes, namely microbiomes with low NSTIs (≲15*%* for the samples examined here), although tools occasionally disagreed substantially even on samples with low NSTIs. We note that here, we recovered OTUs by closed-reference clustering to SILVA, thereby omitting clades not represented in SILVA at all. It is likely that many of these omitted clades, especially those from poorly studied phyla, had even greater NSTDs than typical closed-reference OTUs. This realization further strengthens our conclusions that existing GCN prediction tools perform poorly for many of those samples.

Previous studies have used mock communities to test the predictability of 16S GCNs, demonstrating that correcting for GCNs improves estimates of microbial community composition, provided that corrections are accurate [6, 7, 22]. While mock communities using cultured and sequenced strains are convenient test cases (since GCNs are known for each member), they lead to a biased assessment of predictive accuracy because strains used in mock communities are likely to be found among (or closely related to) sequenced genomes. In other words, use of mock communities — instead of natural communities as in the present study — can yield the false impression that GCNs can be well predicted for typical natural microbial communities.

## Implications

Accurate correction of 16S sequence variant counts for GCNs in microbiome surveys would undoubtedly reduce biases in cell-count estimates. As we have shown, however, predicting 16S GCNs can come at a cost of substantial additional errors (“noise”) when affected clades do not have close relatives with sequenced genomes. These errors can even vary strongly between tools (Figs. 3 and 4). In principle, GCN corrections may be applied selectively to only those taxa with a sufficiently low NSTD, although this complicates the interpretation of microbial community profiles that include taxa with no GCN correction. Adoption of GCN corrections, dependent or independent of NSTDs, as suggested by other authors [5, 7], could thus compromise the comparability between studies. We recommend a careful consideration of these caveats before correcting for GCNs in typical microbiome studies, until coverage by sequenced genomes is substantially improved. A similar conclusion was recently reached by Edgar [22], based on tests of a specific GCN correction algorithm on mock microbial communities. For example, if the detection of spatiotemporal variation in community composition is the sole objective in a study, then this variation could be described merely in terms of 16S gene variants without the need for normalizing by GCNs. A notable exception are cases that necessitate knowledge of true OTU proportions in a community, such as for biogeochemical modeling or for estimating gene proportions in a community using tools such as PICRUSt [6] or PAPRICA [8]. In these cases, it may be reasonable to correct for 16S GCNs despite the high additional noise, although the effects of this noise on estimated gene abundances remain to be investigated. Recent tools such as “16Stimator” could help extend coverage to de novo assembled draft genomes [23], for which GCN counts have been hard to estimate in the past due to misassembly of ribosomal genes. Our findings also point to the need to explore alternative genes with more conserved copy numbers for phylogenetic community profiling, such as *recA* or *rpoB* [24, 25]. Larger reference databases for phylogenetic identification of sequence variants of these genes are needed, however, to make these genes a more widely adopted alternative to 16S rRNA.

More generally, our work demonstrates the importance of a cautious interpretation of evolutionary statistics (in this case, Blomberg’s *K* and Pagel’s *λ* [5, 7]) to avoid hasty conclusions about the predictability of a trait using phylogenetic methods. Indeed, two of the most important factors influencing the predictability of a trait is the phylogenetic distance to the nearest clade with known trait value (in our case, a sequenced genome) and the typical depth at which the trait is conserved [10, 26]. Unitless statistics such as *K* and *λ* indicate that a phylogenetic signal exists but say little about the two factors that are crucial for accurately predicting traits based on phylogeny in practice. Similar considerations are warranted when phylogenetic extrapolation tools such as PICRUSt [6] or PAPRICA [8] and interpolation tools such as FAPROTAX [27] are used to estimate metabolic traits from phylogeny. Indeed, Langille et al. [6] emphasize that the accuracy of PICRUSt depends on the availability of closely sequenced relatives. We point out that phylogenetic conservatism varies strongly across traits [28, 29], and thus, our ability to accurately predict these traits using phylogenetic methods also varies considerably.

## Conclusions

Here, we have assessed the phylogenetic conservatism of 16S GCNs and examined the predictability of GCNs using several common phylogenetic reconstruction algorithms, as a function of a clade’s nearest sequenced taxon distance. Our findings suggest that GCNs may currently not be predictable for a substantial fraction of extant prokaryotic clades. Further, we independently evaluated the accuracy of available 16S GCN prediction tools [6–8] on a set of completely sequenced genomes, as well as for OTUs in the Greengenes 16S database and in microbial communities from a wide range of environments. Our analysis revealed that existing tools perform poorly for a large fraction of the genomes and OTUs tested. For over 85% of the examined microbial communities, GCN predictions differed strongly between any two tools compared (*R*^2^<0.5). Thus, contrary to common assumption, 16S GCN predictions are currently bound to be inaccurate for a substantial fraction of extant prokaryotic diversity due to insufficient coverage by sequenced genomes. We therefore recommend that 16S GCNs should only be corrected for in surveys of microbial communities with a low NSTI (≲15*%*), unless the high additional noise introduced is justified by a need to estimate true cell proportions.

## Materials and methods

### Construction of SILVA-derived tree

While the original SILVA tree is well curated taxonomically, it is mostly meant to be used as a guide tree, and re-calculation of branch lengths is generally advised for downstream phylogenetic analyses [30]. Here, to construct a phylogenetic tree with more meaningful branch lengths using OTUs in the SILVA non-redundant (NR99) 16S database (release 128; [4]), we proceeded as follows. Aligned representative SSU sequences in SILVA were reduced by first removing nucleotide positions with >95*%* gaps and then removing the top 5% most entropic nucleotide positions. Taxonomic identities provided by SILVA for OTUs at the domain, phylum, and class level were used to create split constraints for FastTree [31], by constraining each taxon to be on a single side of a split and monophyletic. Taxa with fewer than 10 OTUs were omitted from the constraints. A total of 354 constraints were thus defined. Using the taxonomically generated constraints and taking the original SILVA tree as a starting tree, we constructed a phylogenetic tree from the reduced alignments with FastTree v2.1.10 (options “-spr 4 -gamma -fastest -no2nd -constraintWeight 100”). The phylogenetic tree was rerooted so that bacteria and Archaea are split at the root. Our SILVA-derived tree is provided as Additional file 2. For all downstream analyses, chloroplasts, mitochondria, and Eukaryota were omitted from the tree. In the main article, we describe our analyses using this SILVA-derived tree (Fig. 1); analogous results for the original SILVA tree are shown in Additional file 1: Figure S1.

### Phylogenetic distribution of 16S GCNs

To examine how 16S GCNs are distributed phylogenetically and to assess their general predictability using various phylogenetic methods, we proceeded as follows. A total of 8,767 annotated bacterial and archaeal genomes with completion status “Complete Genome” were downloaded from the NCBI RefSeq database on January 4, 2018. Downloaded genomes were checked for potential contamination using checkM 1.0.6 [32] (option “reduced_tree”), which is based on the detection of conserved marker genes (assembly and checkM summaries in Additional file 3). Genomes found to exhibit a contamination level above 1% or a strain heterogeneity above 1% were discarded, leaving us with 6,868 complete genomes for downstream analysis (Additional file 4).

For each genome, 16S GCNs were determined using two approaches: First, we counted the number of annotated 16S rRNA sequences in the NCBI annotations (files rna_from_genomic.fna). Second, we used covariance models with the program cmsearch (as part of INFERNAL version 1.1.2, options “--noali --cut_nc”) to search for 16S rRNA sequences within the assembled genomes (files genomic.fna). Separate covariance models for archaeal and bacterial 16S rRNA genes were obtained from the Rfam database [33] (accessions RF00177 and RF01959). A table listing GCNs calculated using both methods is provided as Additional file 5. Only genomes for which the two methods yielded the same 16S GCNs were considered for subsequent analysis, yielding 16S GCNs for 6,780 genomes (“high-quality genomes,” Additional file 6). The accuracy of these GCNs was further verified through comparison to the Ribosomal RNA Operon Copy Number Database (rrnDB, accessed on June 7, 2017; [34]) whenever a genome assembly accession was present in the rrnDB (rrnDB attribute “Data source record id”). Across 5,616 high-quality genomes tested, we found an almost-perfect agreement with the rrnDB (*R*^2^>0.999; Additional file 1: Figure S2). checkM quality summaries for the high-quality genome set are provided as Additional file 7.

Tips on the SILVA-derived tree were mapped to high-quality genomes, whenever possible, as follows: First, representative 16S sequences of SILVA OTUs were aligned to the longest 16S rRNA sequence from each genome using vsearch 2.3.4 [35] at maximum (100%) similarity (vsearch options “--strand both --usearch_global --maxaccepts 0 --top_hits_only --iddef 0 --id 1.0”). If an OTU aligned to multiple genomes, all genomes were initially kept. Next, for each aligned OTU-genome pair, we compared the NCBI taxon ID (“taxid”) of the OTU to that of the genome. OTU taxids were obtained from a lookup table provided by SILVA (https://www.arb-silva.de/fileadmin/silva/_databases/release/_128/Exports/taxonomy/taxmap/_embl/_ssu/_ref/_128.txt.gz). Genome taxids were obtained from lookup tables provided by NCBI (ftp://ftp.ncbi.nlm.nih.gov/genomes/genbank/*/assembly_summary.txt, where “*” is either “bacteria” or “archaea”). Any aligned OTU-genome pair with non-identical taxids was omitted. Of the remaining OTU-genome pairs with identical taxids, we only kept the first aligned genome for each OTU. A total of 9,395 OTUs could thus be mapped to one of the genomes. For each mapped OTU, we assumed a GCN equal to the GCN counted for the corresponding genome. For all other OTUs, we assumed an unknown GCN.

All phylogenetic analyses were performed using the R package castor [36], available at The Comprehensive R Archive Network (CRAN). NSTDs for all tips with respect to tips mapped to a sequenced genome (Fig. 1b) were calculated using the castor function find_nearest_tips. The phylogenetic autocorrelation function (ACF) of known 16S GCNs across the SILVA-derived tree (Fig. 1a) was calculated using the castor function get_trait_acf based on 10^8^ tip pairs (options “Npairs=1e8, Nbins=100”), chosen randomly among tips with known GCN. The function get_trait_acf randomly picks OTU pairs on the tree, bins them into one of many intervals of phylogenetic distance, and calculates the Pearson autocorrelation between GCNs of the OTU pairs within each bin. Note that this analysis does not assume that GCNs scale linearly with phylogenetic distance. Instead, the ACF merely measures the statistical correlation between GCNs on distinct tips, conditional upon the tips being within a certain phylogenetic distance from each other.

GCNs were reconstructed on the SILVA-derived tree using Sankoff’s maximum-parsimony (function hsp_max_parsimony, with option transition_costs either set to “exponential,” “proportional,” or “all_equal”), phylogenetic independent contrasts (function hsp_independent_contrasts), weighted-squared-change parsimony (function hsp_squared_change_parsimony), subtree averaging (function hsp_subtree_averaging), and maximum-likelihood of Mk models with rerooting (function hsp_mk_model_rerooting with options root_prior=‘empirical’, optimization_algorithm=‘nlminb’, Ntrials=5, rate_model=‘ER’).

To calculate the cross-validated fraction of variance predicted by (aka. cross-validated coefficient of determination of) each method ($R^{2}_{\text {cv}}$; [17]) as a function of the NSTD (Fig. 1c), we proceeded as follows. We randomly chose 2% of the tips with known 16S GCN to be excluded from the input to the reconstructions and to be used as an independent “test set” afterwards. Depending on the NSTD cutoff considered (for example 10% substitutions per site), we also excluded all tips whose phylogenetic distance to the test set was below the NSTD cutoff. The remaining tips with known GCNs (“training set”) were used as input to the reconstructions, and the GCNs predicted for the test set were then compared to the known GCNs of the test set. This process was repeated three times and the resulting *R*^2^ was averaged over all repeats, yielding an $R^{2}_{\text {cv}}$ for each considered NSTD cutoff. The R script for analyzing and reconstructing 16S GCNs across the SILVA-derived tree is available as Additional file 8. For comparison, all of the above analyses were also performed using the original SILVA guide tree (Additional file 1: Figure S1).

### Evaluation of 3rd party GCN prediction tools on sequenced genomes

To test the predictive accuracy of CopyRighter [7], PICRUSt [6], and PAPRICA [8] for genomes with known GCNs, we compared their predictions with the GCNs counted in the (high-quality) sequenced genomes. To evaluate the predictive accuracy of CopyRighter [7] on the genomes, we proceeded as follows: We first downloaded the precomputed lookup table listing CopyRighter’s predictions for the Greengenes 16S rRNA database (release October 2012, “GG2012”; [20]), from the project’s Github on June 6, 2017 (v0.46): https://github.com/fangly/AmpliCopyRighter (CopyRighter-0.46/data/201210/ssu_img40_gg201210.txt). We then aligned the longest 16S rRNA sequence of each genome to OTUs (clustered at 99% similarity) in the Greengenes database using vsearch (vsearch options “--strand both --usearch_global --maxhits 1 --maxaccepts 10 --top_hits_only”), always choosing the best match in Greengenes and keeping only genomes that mapped to a Greengenes entry by at least 99% similarity (5688 genomes mapped). For each mapped genome, we took the GCN predicted by CopyRighter for the corresponding Greengenes entry as CopyRighter’s prediction for the genome. This prediction was then compared to the GCN counted from the genome sequence. A histogram of CopyRighter’s predictions across mapped genomes is shown in Additional file 1: Figure S4B. The predictive accuracy of CopyRighter was measured in terms of the fraction of explained variance (*R*^2^), as a function of a genome’s NSTD (Fig. 1a). NSTDs of genomes were calculated as described in a separate section below.

A similar approach was used for PICRUSt [6]: The precomputed lookup table listing PICRUSt’s predictions for the Greengenes database (release May 2013; “GG2013”) was downloaded from the project’s website on June 6, 2017 (v1.1.1): https://picrust.github.io/picrust/picrust/_precalculated/_files.html (16S_13_5_precalculated.tab.gz). A total of 5,708 high-quality genomes could be mapped to an OTU (99% similarity) in GG2013. A histogram of PICRUSt’s predictions across all mapped genomes is shown in Additional file 1: Figure S4C. The predictive accuracy of PICRUSt was measured in terms of the *R*^2^ as a function of a genome’s NSTD (Fig. 1b), similarly to CopyRighter.

To evaluate the predictive accuracy of PAPRICA [8] on the genomes, we proceeded as follows: We first downloaded and installed PAPRICA from the project’s Github on June 6, 2017 (v0.4.0b): https://github.com/bowmanjeffs/paprica. This release includes precomputed reference trees (one for archaea and one for bacteria) and tables listing 16S GCNs for the tool’s calibration genomes represented in the reference trees. We used the longest 16S rRNA sequence from each genome as an input to the PAPRICA pipeline (command “paprica-run.sh”), separately for archaea and bacteria. The pipeline produces, among others, a table listing the uncorrected abundance of each unique input sequence (this can be greater than 1 if multiple genomes share the same 16S rRNA sequence) and the corresponding corrected abundance (after dividing by the predicted 16S GCN). We used this table to obtain the 16S GCNs predicted by PAPRICA for the unique 16S sequences (representing 3473 16S sequences), by dividing the uncorrected by the corrected abundance. We then compared these predicted GCNs to the GCNs counted in the genome sequences, similarly to above. A histogram of PAPRICA’s predictions across all represented genomes is shown in Additional file 1: Figure S4D. The predictive accuracy of PAPRICA was measured in terms of the *R*^2^ as a function of a genome’s NSTD (Fig. 1a), similarly to CopyRighter.

### Comparison of 3rd party GCN prediction tools across Greengenes

To compare the predictions by CopyRighter to those by PICRUSt across all OTUs in Greengenes (Fig. 3a), we first mapped all OTUs in GG2013 to OTUs in GG2012 using vsearch (with options “--strand both --usearch_global”). We only kept matches at 100% similarity (153,375 out of 203,452 OTUs in GG2013). To each mapped OTU in GG2013, we compared the corresponding GCN predicted by PICRUSt to the GCN predicted by CopyRighter for the matched OTU in GG2012. To calculate the frequency distributions of GCNs predicted by CopyRighter and PICRUSt across all OTUs in Greengenes (histograms in Additional file 1: Figure S3A,B), we used the GCNs listed in their precomputed lookup tables.

To compare PAPRICA to PICRUSt across Greengenes (Fig. 3b), we proceeded as follows: Representative sequences of OTUs in GG2013 were split into archaeal and bacterial sequences. Each resulting fasta file was used as input to the PAPRICA pipeline to predict the corresponding 16S GCN, as described above for genomes. This yielded a predicted GCN for all Greengenes entries. These predictions were compared to the precomputed GCN values provided by PICRUSt. These predictions were also used to calculate the frequency distribution of GCNs predicted by PAPRICA across Greengenes (Additional file 1: Figure S3C). To compare CopyRighter to PAPRICA (Fig. 3c), we proceeded as described above for the comparison of CopyRighter to PICRUSt.

### Comparison of 3rd party GCN prediction tools across microbial communities

To compare CopyRighter, PICRUSt, and PAPRICA across OTUs in various microbial communities, we proceeded as follows. Publicly available 16S rRNA amplicon sequence data from various environmental samples were downloaded from the European Nucleotide Archive (http://www.ebi.ac.uk/ena). Only Illumina sequence data from amplicons obtained using bacteria- and/or archaea-sensitive primers were considered. Samples were chosen to cover a wide range of environments, including the ocean, marine and lake sediments, soil, saline and hypersaline lakes, hydrothermal vents, hot springs, bioreactors, and animal-associated microbiomes. All sequencing data were processed in a similar way, where possible, as follows. Overlapping paired-end reads were merged using flash v1.2.11 [37] (options –min-overlap=20 –max-overlap=300 –max-mismatch-density 0.25 –phred-offset=33 –allow-outies), and non-overlapping paired-end reads were omitted. Single-end reads were kept unchanged. All single-end reads and merged paired-end reads were then quality filtered using vsearch v2.4.3 [35] (options –fastq_ascii 33 –fastq_minlen 120 –fastq_qmin 0 –fastq_maxee 1 –fastq_truncee 1 –fastq_maxee_rate 0.005 –fastq_stripleft 7). Samples with more than 20,000 quality-filtered reads were rarefied down to 20,000 reads to reduce computation time, by randomly picking reads without replacement. Quality-filtered sequences were clustered into operational taxonomic units (OTUs; at 97% similarity) by closed-reference global aligning against the non-redundant (NR99) SILVA SSU reference database (release 128; [4]), using vsearch. Both strands were considered for alignment (vsearch option --strand both). Sequences not matching any database entry at 97% similarity or higher were discarded. Note that OTUs were thus represented by SILVA entries, namely the ones used to seed the clusters. Chloroplasts, mitochondria, and any Eukaryota were omitted. OTUs represented by fewer than five reads across all samples were omitted. Finally, any samples with fewer than 2,000 reads accounted for by OTUs were omitted. This yielded an OTU table with 635 samples and 65,673 OTUs represented by 4,827,748 reads (on average 734 OTUs per sample). Sample accession numbers, coordinates, sampling dates, original publications, sequencing platforms, quality-filtered read lengths, and read counts and covered primer regions (where available) are provided in Additional file 9.

To predict GCNs for OTUs in each sample using CopyRighter, we used the same approach as for genomes: Representative 16S sequences of OTUs were aligned to GG2012 using vsearch (options “--strand both --usearch_global --iddef 0 --id 0.99 --maxhits 1 --maxaccepts 10 --top_hits_only”), omitting any OTUs not matched to a Greengenes entry by at least 99% similarity. For each OTU kept, the GCN listed by CopyRighter for the matched Greengenes entry was taken as CopyRighter’s prediction. For PICRUSt, we proceeded in an analogous way, using GG2013 instead of GG2012. For PAPRICA, we proceeded in an analogous way, using PAPRICA’s GCN predictions computed previously for GG2013 (see previous section).

To compare any two given tools (CopyRighter vs. PICRUSt, PICRUSt vs. PAPRICA, or CopyRighter vs. PAPRICA) for a specific sample, only OTUs with at least one read in the sample and having a GCN prediction from both tools were considered. We measured the agreement between two tools in terms of the fraction of variance in predictions of the 1st tool that was explained by predictions of the 2nd tool (*R*^2^). We calculated the sample’s NSTI (nearest sequenced taxon index) according to [6], i.e., as the arithmetic average NSTD over all OTUs considered in the comparison and weighted by relative OTU frequencies. Details on how NSTDs were calculated are provided in the section below. For each pair of tools compared, we thus obtained 635 NSTIs and 635 *R*^2^s across 635 samples, shown in Fig. 4. Pearson correlation coefficients (*r*^2^) between NSTIs and *R*^2^ were calculated for each pair of tools, separately for animal-associated and non-animal-associated samples. Statistical significances (*P* values) of correlation coefficients were estimated using a permutation test with 1000 permutations. Additional file 1: Figures S6 and S7 show GCNs predicted by each tool for various microbial communities. We also show relative deviations between tools (|*A*−*B*|/((*A*+*B*)/2), where *A* and *B* are GCNs predicted by two tools for the same OTU) and NSTDs for OTUs in various samples (Additional file 1: Figure S8).

### Evaluation and comparison of GCN prediction tools depending on NSTD

To examine the predictive accuracy of CopyRighter, PICRUSt, and PAPRICA as a function of an OTU’s or genome’s NSTD, we proceeded as follows. For each OTU in SILVA, and separately for each tool, we calculated the NSTD as the phylogenetic distance to the nearest sequenced genome used by the tool to make predictions (“calibration genomes”). For PAPRICA, a list of 5,628 calibration genomes was obtained from PAPRICA’s precomputed files (PAPRICA/ref_genome_database/*/genome_data.final.csv, where “*” is either bacteria or archaea). Calibration genomes were matched to SILVA OTUs via global alignment of the 16S gene at a similarity threshold of 99%, using vsearch. Matched OTUs were assumed to have an NSTD equal to zero, and for all other SILVA OTUs, the NSTD was calculated based on the SILVA-derived tree and using the R package castor [36]. An approximate matching of genomes to OTUs (i.e., at 99% similarity) was chosen to ensure that as many of the calibration genomes are included as possible; note that SILVA OTUs are themselves clustered at that similarity and that the error potentially introduced to the NSTDs and NSTIs is negligible (< 1% nucleotide substitutions per site). For PICRUSt, a table was downloaded from the project’s website listing IMG (Integrated Microbial Genomes) IDs for 2,887 calibration genomes (https://github.com/picrust/picrust/tree/master/tutorials/picrust/_starting/_files.zip, file GG_to_IMGv350.txt). IMG IDs were translated to GG2013 sequence IDs using the gg_13_5_img.txt lookup table downloaded from the Greengenes website (http://greengenes.secondgenome.com/downloads). Matched GG2013 IDs were then mapped to SILVA OTUs via global 16S sequence alignment with vsearch, at a similarity threshold of 99%. NSTDs of SILVA OTUs were then calculated in the same way as for PAPRICA. For CopyRighter, a lookup table was downloaded from the project’s Github page that maps calibration genomes to GG2012 sequences (https://github.com/fangly/AmpliCopyrighter, file AmpliCopyrighter-0.46/preprocessing/ data/img_to_gg.txt). GG2012 sequences listed in that table were mapped to SILVA OTUs, and NSTDs were calculated for all SILVA OTUs, in a similar way as for PICRUSt. To determine the NSTDs for genomes examined in this study (separately for CopyRighter, PICRUSt, and PAPRICA), genomes were mapped to SILVA OTUs via global alignment of their longest available 16S sequence at 99% similarity. For each genome, the NSTD of the most closely matched SILVA OTU was taken as the genome’s NSTD. To determine NSTDs for all Greengenes OTUs, we mapped Greengenes OTUs to SILVA OTUs via global alignment at 99% similarity. To determine NSTDs for OTUs recovered from the sampled microbial communities, we directly used the NSTDs of SILVA OTUs used as seeds during closed-reference OTU picking. When comparing two GCN prediction tools on an OTU (e.g., Figs. 3 and 4 and Additional file 1: Figure S8), in cases where the two NSTDs differed, we used their arithmetic average. To calculate the *R*^2^ between any two GCN prediction tools, or between a GCN prediction tool and the “true GCNs,” as a function of the NSTD (Figs. 2 and 3d–f), we binned the OTUs or genomes used in the comparison into equally sized NSTD intervals and calculated the *R*^2^ separately for each interval. Only NSTD intervals with at least 10 OTUs or genomes were considered.

## Additional files

Additional file 1 Supplementary Information. (PDF 4320 kb)

Additional file 2 SILVA-derived 16S tree (NR99). (TRE 23800 kb)

Additional file 3 Assembly stats for all genomes. (TSV 13200 kb)

Additional file 4 Assembly stats for quality-filtered genomes. (TSV 910 kb)

Additional file 5 16S GCNs for quality-filtered genomes using both methods. (TSV 135 kb)

Additional file 6 16S GCNs counted for high-quality genomes. (TSV 120 kb)

Additional file 7 Assembly stats for high-quality genomes. (TSV 898 kb)

Additional file 8 R script for phylogenetic analysis of 16S GCNs on SILVA. (R 34.3 kb)

Additional file 9 Metadata for microbiome samples. (TSV 127 kb)

Additional file 10 16S GCNs for SILVA OTUs matched to high-quality genomes. (TSV 228 kb)

Additional file 11 16S GCNs predicted for SILVA 16S guide tree (NR99) via MPR.exp. (TSV 15200 kb)

Additional file 12 16S GCNs predicted for SILVA-derived 16S tree (NR99) via MPR.exp. (TSV 14700 kb)

## Abbreviations

GCN: Gene copy number
NSTI: Nearest sequenced taxon index
NSTD: Nearest sequenced taxon distance
