# Supplementary material for: Correcting for 16S rRNA gene copy numbers in microbiome surveys remains an unsolved problem
Source: Microbiome. 2018 Feb 26;6:41. doi: 10.1186/s40168-018-0420-9 (PMC5828423; doi:10.1186/s40168-018-0420-9)
Supplement: Supplementary file 1 — Supplementary Information. (PDF 4320 kb) [file 40168_2018_420_MOESM1_ESM.pdf]

Correcting for 16S rRNA gene copy numbers in microbiome surveys remains an unsolved problem
# - Supplementary Information -

Stilianos Louca[1,2,*], Michael Doebeli[1,2,3] & Laura Wegener Parfrey[1,2,4]

[1]*Biodiversity Research Centre, University of British Columbia, Canada*
[2]*Department of Zoology, University of British Columbia, Canada*
[3]*Department of Mathematics, University of British Columbia, Canada*
[4]*Department of Botany, University of British Columbia, Canada*
[*]Corresponding author: louca.research@gmail.com

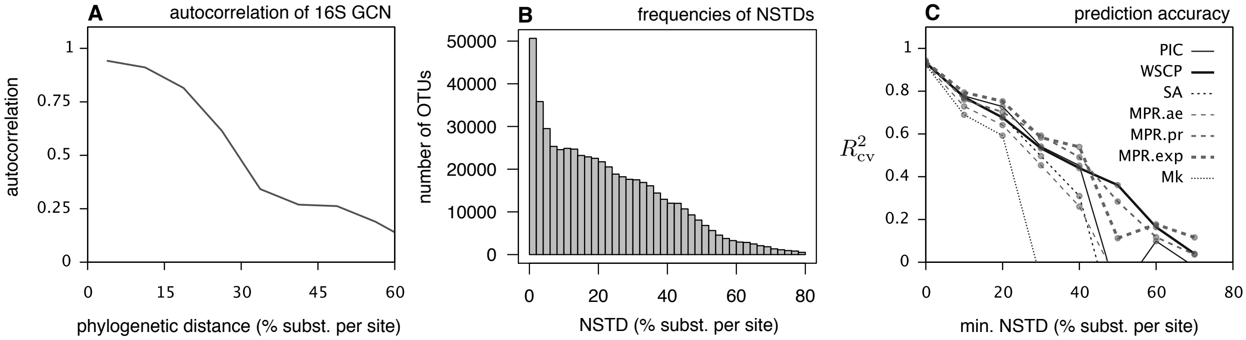

Figure S1: **Phylogenetic signal of 16S gene copy numbers (original SILVA tree).** (A) Pearson autocorrelation function of 16S GCNs depending on phylogenetic distance between tip pairs, estimated based on ∼6,800 sequenced genomes. (B) Distances of tips in the SILVA tree to the nearest sequenced genome. Each bar spans an NSTD interval of 2%. (C) Cross-validated coefficients of determination (R^2_{cv}) for 16S GCNs predicted on the SILVA tree and depending on the minimum NSTD of the tips tested, for various ancestral state reconstruction algorithms (PIC: phylogenetic independent contrasts, WSCP: weighted squared-change parsimony, SA: subtree averaging, MPR: maximum parsimony reconstruction, Mk: continuous-time Markov chain model with equal-rates transition matrix). MPR transition costs either increased exponentially with transition size ("exp"), proportionally to transition size ("pr") or were equal for all transitions ("ae").

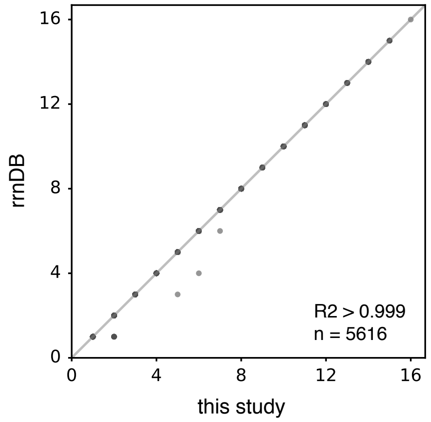

Figure S2: **Comparisons of 16S GCNs calculated for genomes by this study and the rrnDB.** 16S GCN estimates provided by rrnDB (Stoddard *et al.*, 2014) compared to GCNs counted for genomes in this study ("high-quality genome set"). One point per genome. The diagonal line is shown for reference. The fraction of explained variance (R^2, X-axis explaining Y-axis) and the number of genomes (n) are written in the figure.

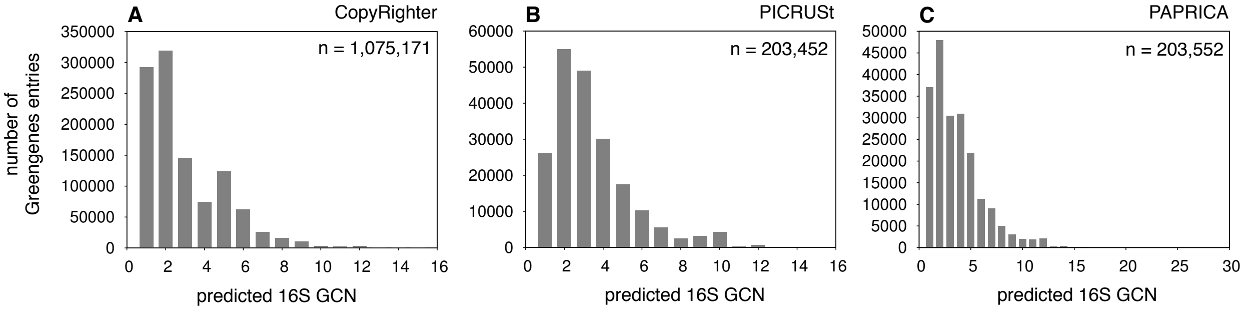

Figure S3: **Predicted 16S GCN frequency distributions across Greengenes.** Frequency distributions of 16S GCNs predicted by CopyRighter (A), PICRUSt (B) and PAPRICA (C) across the Greengenes 16S rRNA reference database (release October 2012 for CopyRighter, release May 2013 for PICRUSt and PAPRICA; McDonald *et al.*, 2012). For CopyRighter and PICRUSt, frequency distributions were calculated directly from the precomputed tables obtained from each project's website (see Methods). In (C), representative sequences of OTUs (99% similarity) in Greengenes (release May 2013) were used as input to PAPRICA. Sample sizes (n) are written inside the figures.

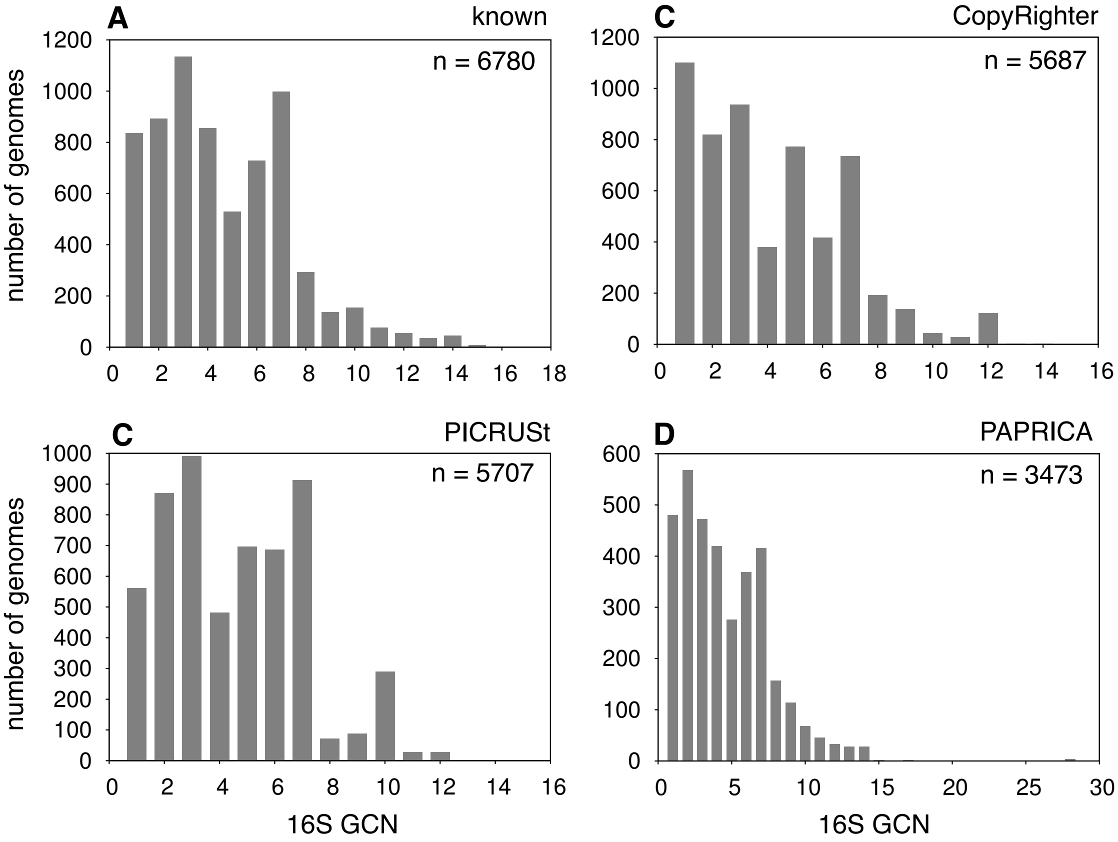

Figure S4: **16S GCN frequency distributions across genomes.** Frequency distributions of 16S GCNs across sequenced genomes, known based on the genome sequence (counted in this study) (A), as well as predicted by CopyRighter (B), PICRUSt (C) and PAPRICA (D) using phylogenetic methods. Sample sizes (n) are written in each figure. Precise genome subsets differ between tools due to methodological constraints. Non-integer GCN predictions were rounded to the nearest integer.

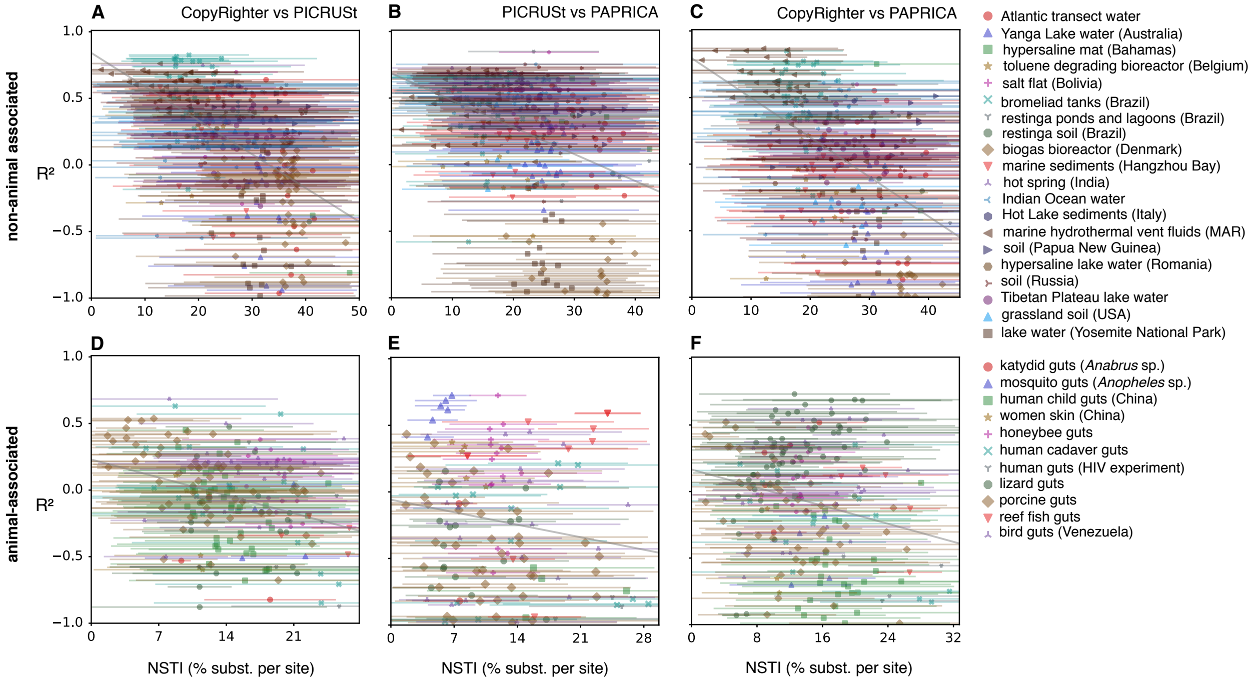

Figure S5: **Agreement of GCN prediction tools in microbial communities, depending on the NSTI.** (A) Agreement between 16S GCNs predicted by CopyRighter and PICRUSt (in terms of the fraction of variance in the former explained by the latter, R^2) for non-animal-associated microbial communities, compared to the nearest sequenced taxon index (NSTI, i.e. the weighted mean NSTD) of each community. Each point represents the R^2 and the NSTI of one microbial community sample. Horizontal bars span two (weighted) standard deviations of NSTDs for each sample. (B,C) Similar to (A), but comparing PICRUSt to PAPRICA (B) and CopyRighter to PAPRICA (C). (D–F) Similar to A–C, but for animal-associated samples. In all figures, grey diagonal lines show linear regressions. Pearson correlations between R^2 and NSTI (r^2, written in each figure) were statistically significant ($P < 0.01$) in all cases. Points are shaped and colored according to the original study, as listed in the legend. Apart from the horizontal bars, this figure is the same as Fig. 4 in the main text.

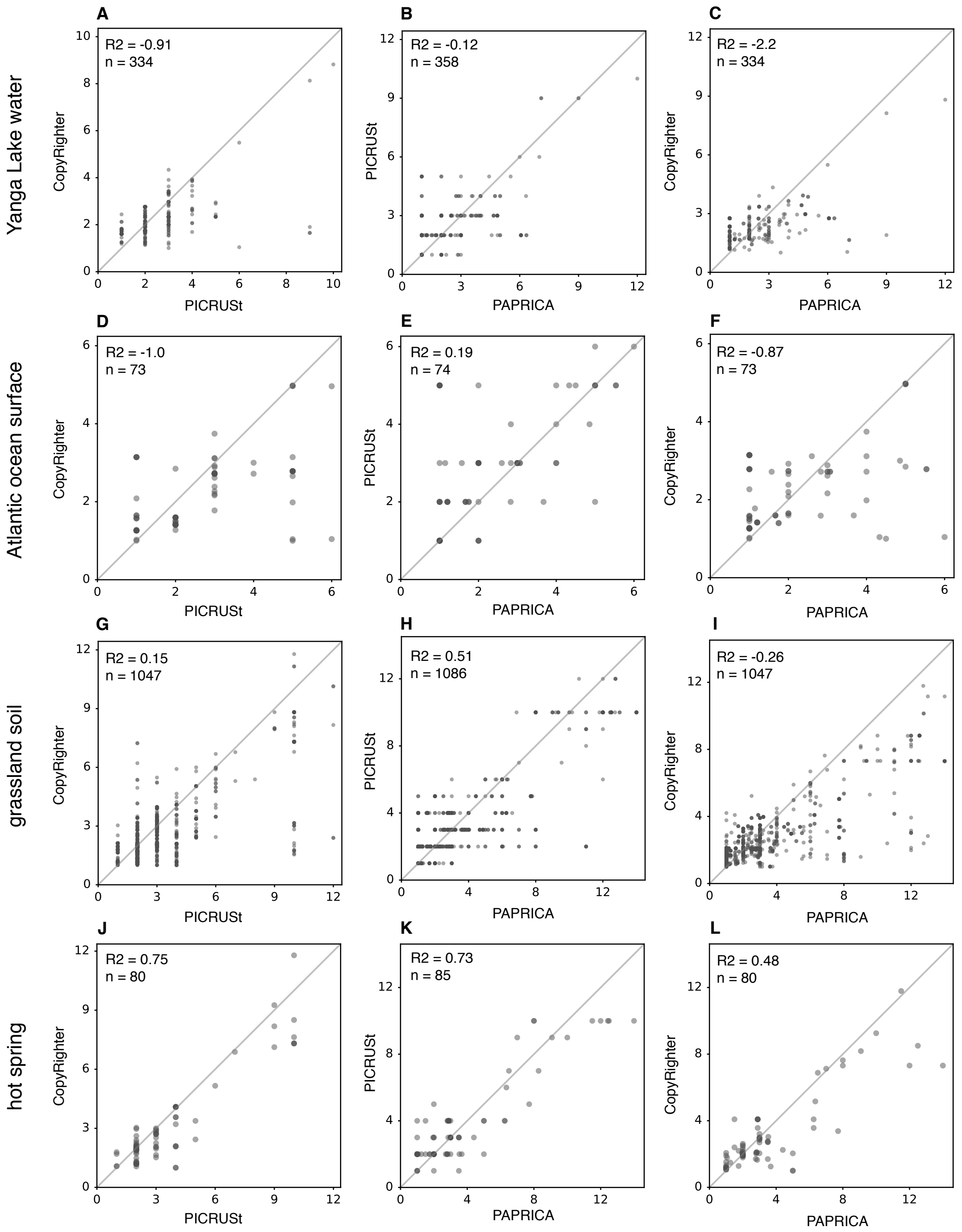

Figure S6: **Comparisons of 16S GCN predictions between tools for microbial communities.** (A–C) 16S GCNs predicted by (A) CopyRighter Angly *et al.*, 2014 and PICRUSt Langille *et al.*, 2013, (B) PICRUSt and PAPRICA (Bowman *et al.*, 2015) and (C) CopyRighter and PAPRICA for prokaryotic OTUs (97% identity) found in a water sample from Yanga Lake, Australia (SRA sample accession SAMN04102871; Woodhouse *et al.*, 2016). One point per OTU. Diagonal lines are shown for reference. Fractions of explained variance (R^2, X-axis explaining Y-axis) and sample sizes (n) are written in each figure. (D–F) Similarly to (A–C), but for prokaryotic OTUs found in an Atlantic ocean surface sample (SAMEA3641572; Milici *et al.*, 2016). (G–I) Similarly to (A–C), but for prokaryotic OTUs found in a USA grassland soil sample (SAMN02746099). (J–L) Similarly to (A–C), but for prokaryotic OTUs found in an Indian hot spring (SAMN03393659; Sahoo *et al.*, 2017). For a comparison of relative deviations between tools and NSTDs, see Fig. S8.

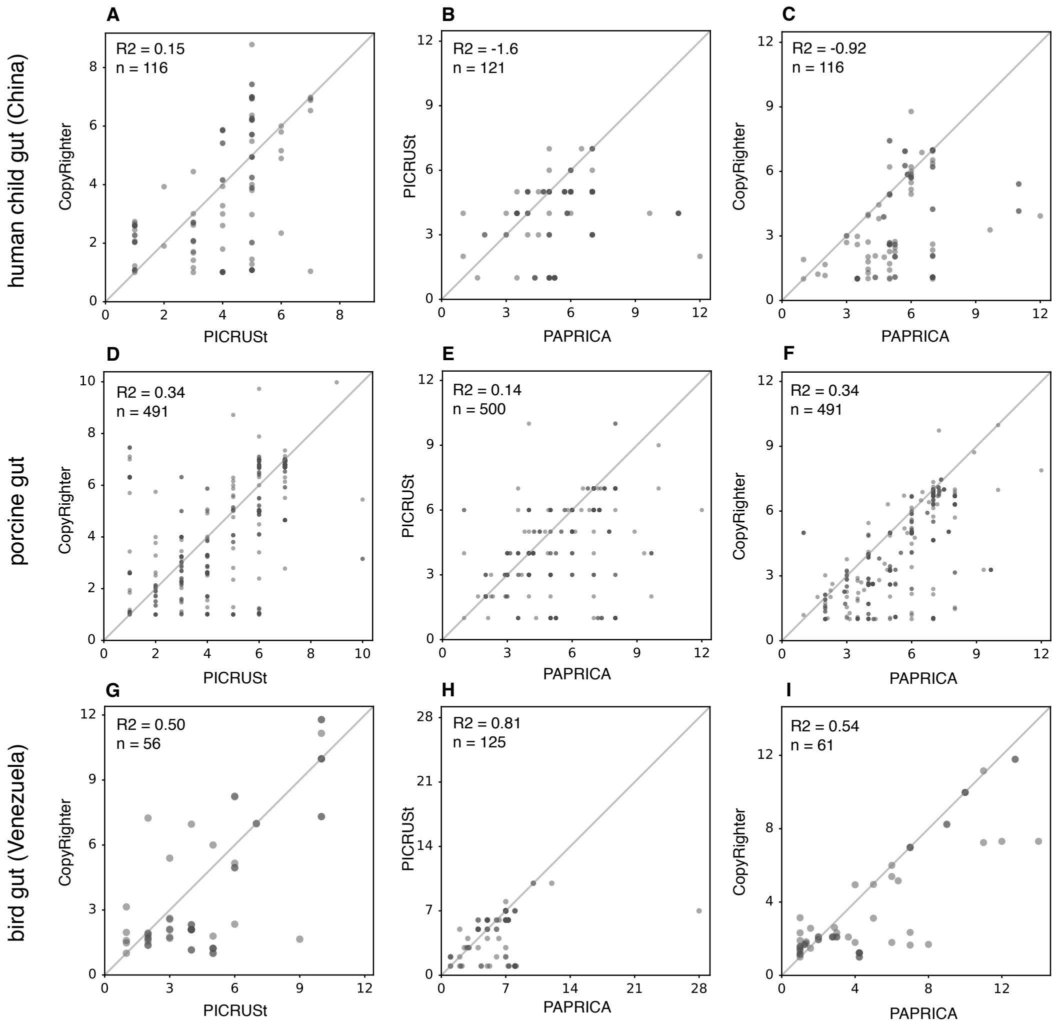

Figure S7: **Comparisons of 16S GCN predictions between tools for animal-associated microbial communities.** (A–C) 16S GCNs predicted by (A) CopyRighter Angly *et al.*, 2014 and PICRUSt Langille *et al.*, 2013, (B) PICRUSt and PAPRICA (Bowman *et al.*, 2015) and (C) CopyRighter and PAPRICA for prokaryotic OTUs (97% identity) found in a human child gut (SRA sample accession SAMN07184108). One point per OTU. Diagonal lines are shown for reference. Fractions of explained variance (R^2, X-axis explaining Y-axis) and sample sizes (n) are written in each figure. (D–F) Similarly to (A–C), but for prokaryotic OTUs found in a porcine gut (SAMN06640712). (G–I) Similarly to (A–C), but for prokaryotic OTUs found in a bird gut (SAMEA4071486). For a comparison of relative deviations between tools and NSTDs, see Fig. S9.

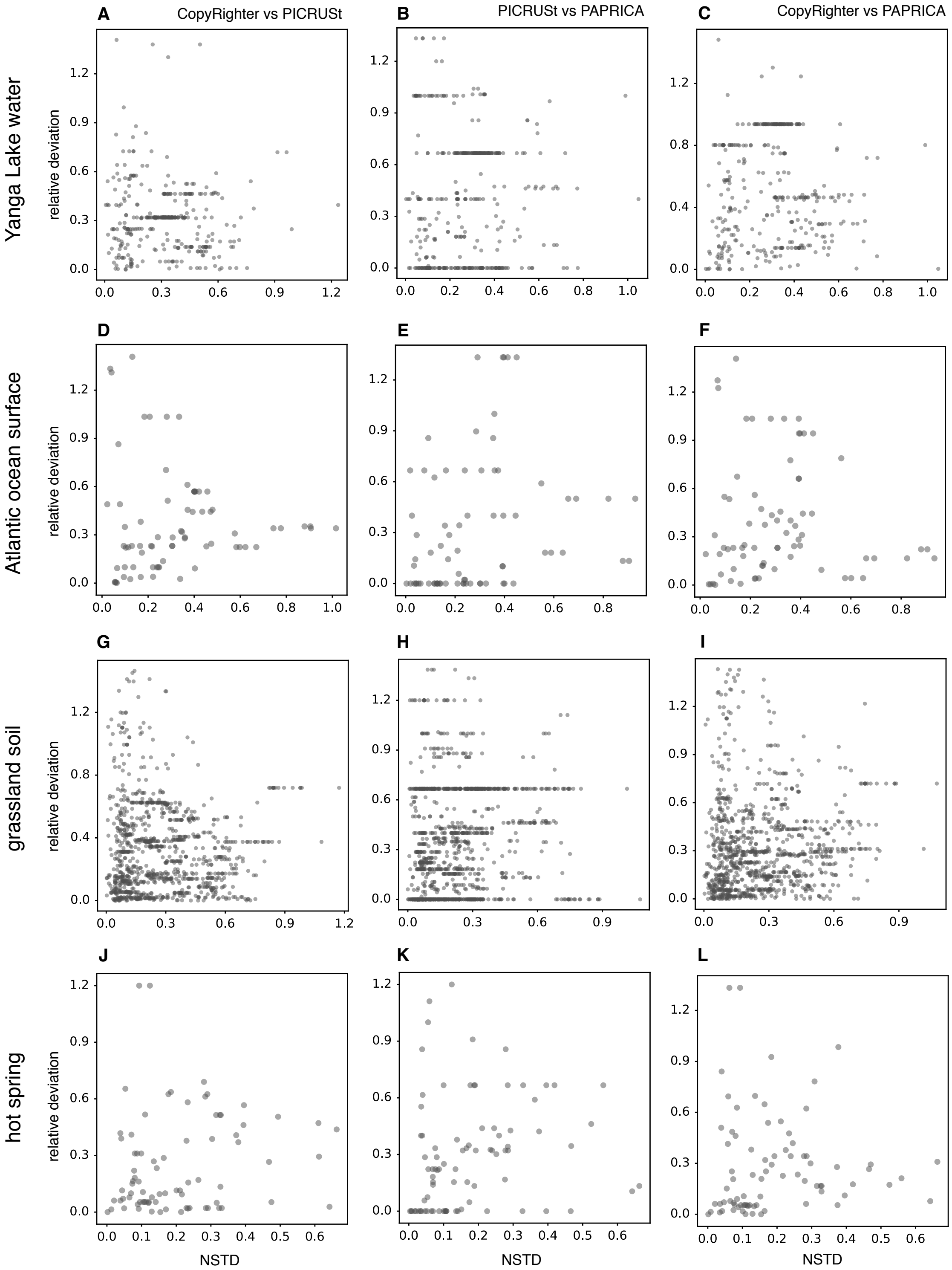

Figure S8: **Relative deviations between 16S GCN predictions, compared to NSTDs in non-animal-associated microbial communities.** Each figure shows relative deviations between GCNs predicted by two tools (vertical axis) compared to NSTDs (horizontal axis) for each OTU in a microbial community (one point per OTU). Left column: Comparing CopyRighter and PICRUSt. Middle column: Comparing PICRUSt and PAPRICA. Right column: Comparing CopyRighter and PAPRICA. Each row shows a different sample (samples as in Fig. S6).

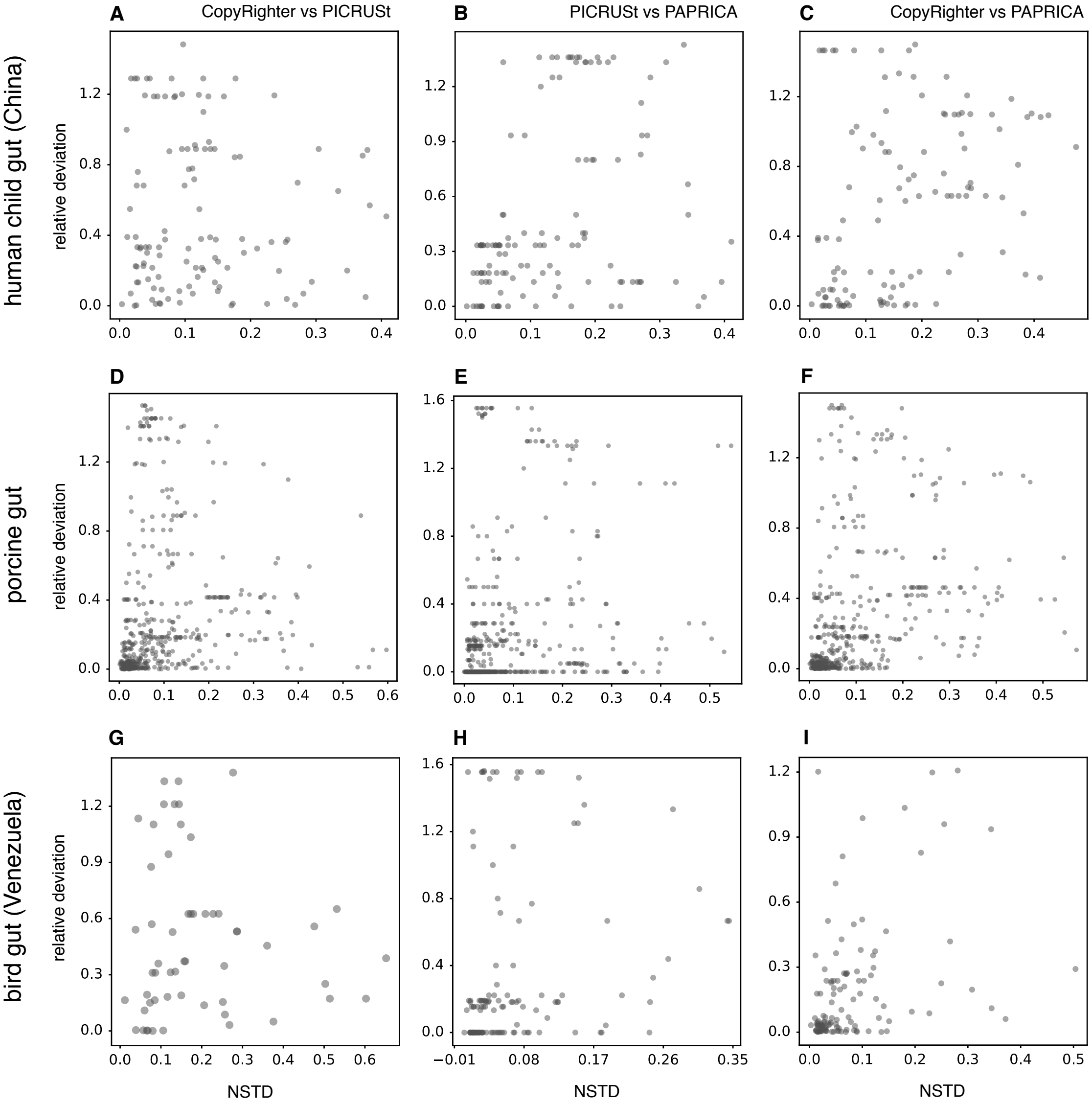

Figure S9: **Relative deviations between 16S GCN predictions, compared to NSTDs in animal-associated microbial communities.** Each figure shows relative deviations between GCNs predicted by two tools (vertical axis) compared to NSTDs (horizontal axis) for each OTU in a microbial community (one point per OTU). Left column: Comparing CopyRighter and PICRUSt. Middle column: Comparing PICRUSt and PAPRICA. Right column: Comparing CopyRighter and PAPRICA. Each row shows a different sample (samples as in Fig. S7).

References

Stoddard, S. F., Smith, B. J., Hein, R., Roller, B. R. & Schmidt, T. M. rrnDB: improved tools for interpreting rRNA gene abundance in bacteria and archaea and a new foundation for future development. *Nucleic Acids Research* **43**, D593–D598 (2014).

McDonald, D. *et al.* An improved greengenes taxonomy with explicit ranks for ecological and evolutionary analyses of bacteria and archaea. *ISME Journal* **6**, 610–618 (2012).

Angly, F. E. *et al.* CopyRighter: a rapid tool for improving the accuracy of microbial community profiles through lineage-specific gene copy number correction. *Microbiome* **2**, 11 (2014).

Langille, M. G. *et al.* Predictive functional profiling of microbial communities using 16S rRNA marker gene sequences. *Nature Biotechnology* **31**, 814–821 (2013).

Bowman, J. S. & Ducklow, H. W. Microbial communities can be described by metabolic structure: A general framework and application to a seasonally variable, depth-stratified microbial community from the coastal west antarctic peninsula. *PLoS ONE* **10**, 1–18 (2015).

Woodhouse, J. N. *et al.* Microbial communities reflect temporal changes in cyanobacterial composition in a shallow ephemeral freshwater lake. *ISME Journal* **10**, 1337–1351 (2016).

Milici, M. *et al.* Low diversity of planktonic bacteria in the tropical ocean. *Scientific Reports* **6**, 19054 (2016).

Sahoo, R. K. *et al.* Comparative analysis of 16S rRNA gene Illumina sequence for microbial community structure in diverse unexplored hot springs of Odisha, India. *Geomicrobiology Journal* **34**, 567–576 (2017).
